# Association Between Specialist Distribution and Regional Variation in Plasmapheresis Use in Japan: A Population‐Level Cross‐Sectional Study

**DOI:** 10.1002/jca.70112

**Published:** 2026-03-15

**Authors:** Ryuichi Yoshimura, Kosuke Matsui, Masahiro Egawa, Yasuko Ito, Kaori Yoshikane, Takafumi Ito

**Affiliations:** ^1^ Division of Nephrology, Department of Internal Medicine Teikyo University Chiba Medical Center Ichihara Japan; ^2^ Division of Nephrology Izumo Medical Life Cooperative Izumo‐Shimin Hospital Izumo Japan

**Keywords:** cross‐sectional study, dialysis, nephrologists, plasmapheresis, population‐level study, regional variation, specialists

## Abstract

Plasmapheresis is widely used in Japan; however, regional variation in its use and its association with specialist distribution remain unclear. We conducted a cross‐sectional study using the 9th National Database Open Data (fiscal year 2022) to assess regional variation in plasmapheresis use and examined its association with the distribution of nephrologists, dialysis specialists, and apheresis specialists. Plasmapheresis use was identified using the relevant receipt code, and age‐ and sex‐adjusted standardized claim ratios (SCRs) were calculated. Plasmapheresis use differed 6.5‐fold across regions, with SCRs ranging from 34.0 to 222.4. Multivariable‐adjusted negative binomial regression models showed that each additional board‐certified nephrologist per 100 000 population was associated with a 1.11‐fold increase in the SCR (*β* = 0.11; 95% confidence interval: 0.02–0.20; *p* = 0.022). No significant associations were observed for board‐certified dialysis or apheresis specialists. These findings indicate substantial regional disparities and suggest that nephrologist distribution may influence plasmapheresis utilization in Japan.

## 1 Introduction

Plasmapheresis, including plasma exchange, double filtration plasmapheresis, and immunoadsorption, is used in diverse medical fields, such as autoimmune, hematologic, renal, neurological, and transplant medicine [1, 2, 3]. Plasmapheresis aims to halt disease progression by removing pathogenic blood plasma components such as autoantibodies, immune complexes, complements, and cytokines [4]. In certain clinical settings, plasmapheresis offers several advantages, including superior clinical outcomes [5, 6, 7], lower healthcare costs [8, 9, 10], and fewer adverse events [11, 12, 13, 14] compared with intravenous immunoglobulin therapy. However, international disparities in plasmapheresis use exist [15], largely influenced by differences in healthcare resources and economic factors [16, 17, 18].

Regional variations have also been observed within individual countries [19, 20, 21]. Among healthcare resources, provider characteristics may contribute to these variations. A study in Australia and New Zealand found that nephrologists had easier access to plasmapheresis than other specialists [22]. However, the potential impact of nephrologist density on plasmapheresis use remains unclear. In Japan, despite widespread clinical use, national utilization patterns and regional variations in plasmapheresis have not been fully explored. Previous Japanese studies have shown regional differences in treatments for asthma [23], cancer [24], and peripheral artery disease [25], which were positively associated with the number of available specialists. These findings suggest that the specialist distribution may influence treatment selection.

We used a national database to assess regional variations in plasmapheresis use across Japan and to examine its association with the numbers of distinct certified specialists involved in apheresis therapy: nephrologists, dialysis specialists, and apheresis specialists.

## 2 Methods

This cross‐sectional study used the National Database (NDB) Open Data Japan [26], which includes over 95% of all health insurance claims in Japan [27]. The 9th NDB Open Data provides fiscal year 2022 claim counts for various medical procedures, including surgeries, medications, and management fees. Data are available in two formats: aggregated by prefecture and stratified by 5‐year age and sex groups for inpatient and outpatient claims. This study was approved by the Ethics Committee of Izumo Medical Life Cooperative Izumo‐shimin Hospital (approval number: 25‐1) and adhered to the principles of the Declaration of Helsinki. Informed consent was obtained via an opt‐out approach in accordance with the Japanese Ethical Guidelines for Medical and Health Research Involving Human Subjects.

### 2.1 Measurement

Plasmapheresis use was identified based on the medical claim code (procedure code: J039; claim code: 140008210), which can be billed once daily when plasmapheresis, including plasma exchange, double filtration plasmapheresis, or immunoadsorption, is performed to remove pathogenic substances.

To account for age and sex distribution differences across prefectures, standardized claim ratios (SCRs) for this code were calculated for all 47 prefectures, as described previously [28, 29]:

$$\mathrm{SCR}=\frac{\text{Observed number of claims}}{\text{Expected number of claims}}$$

An SCR of 100 indicates that a prefecture's age‐ and sex‐adjusted claim count is equivalent to the national average. After summing the number of inpatient and outpatient claims, the expected number of claims in each prefecture was calculated as follows: For each age–sex group (*i*), the prefecture's population (*P*_*i*_) was multiplied by the national claim frequency (*R*_*i*_). The resulting estimates were summed across all age–sex groups to obtain the total expected number of claims:

$$\text{Expected number of claims}=\sum_{i}P_{i}R_{i}$$

The national claim frequency (*R*_*i*_) was calculated by dividing the total national number of claims in each age–sex group by the corresponding national population. These calculations used population data stratified by 5‐year age and sex groups as of October 1, 2022. As these population data were categorized into 36 groups, including age ≥ 85 years, whereas the claim data were categorized into 38 groups, including age ≥ 90 years, the claim data for the 85–89 and ≥ 90 years groups were combined to create 36 age–sex groups. As the NDB Open Data suppresses cells with actual claim counts between 1 and 9, outpatient data were unavailable for two of the 38 age–sex groups, for which only inpatient data were used. Additionally, because total outpatient claim number data were unavailable for three prefectures, their observed number of claims was based solely on inpatient data.

The main independent variable was the number of three specialist types involved in apheresis therapy. Nephrologists, certified by the Japanese Society of Nephrology, must complete ≥ 3 years of training at an education facility designated by the society, in accordance with a specified training curriculum, and provide comprehensive kidney care. Dialysis specialists, certified by the Japanese Society for Dialysis Therapy, must complete ≥ 3 years of training in accordance with the curriculum established by the society and comprehensively manage various blood purification therapies, including apheresis. Apheresis specialists, certified by the Japanese Society for Apheresis, undergo ≥ 5 years of training in apheresis clinical practice and possess the knowledge, skills, and experience necessary to perform apheresis therapy. The number of specialists in each prefecture was obtained as of July 2022 for nephrologists [30], April 2022 for dialysis specialists [31], and October 2022 for apheresis specialists.

The number of hospitals with inpatient capacity for ≥ 20 patients in each prefecture was obtained from the Survey of Medical Institutions as of October 1, 2022 [32]. The number of hospitals per 1000 km^2^ was calculated using land area data on the same date obtained from the Geospatial Information Authority of Japan [33]. The 2022 Basic Survey on Wage Structure provided data on the average monthly wage for general workers by prefecture [34], which were converted to US dollars based on the 2022 average exchange rate (131.454 Japanese yen per US dollar) [35]. Prefecture‐level university enrollment rates were obtained from the 2022 School Basic Survey [36].

### 2.2 Statistical Analyses

All analyses were conducted at the prefecture level. For descriptive purposes, prefectures were categorized into two groups according to the median numbers of board‐certified nephrologists, dialysis specialists, and apheresis specialists to compare clinical characteristics across regions. Characteristic differences between groups were compared using the Wilcoxon rank‐sum test.

Associations between board‐certified specialist numbers and plasmapheresis use were assessed across all 47 prefectures without dichotomization. Negative binomial regression models were applied because the dependent variable exhibited overdispersion inconsistent with Poisson model assumptions [37, 38]. The observed number of plasmapheresis claims was used as the dependent variable. The log‐transformed expected number of claims was included as an offset term to adjust for regional age and sex distribution differences, consistent with the indirect standardization method used for the standardized mortality ratio [39] and standardized incidence ratio [40]. Regression coefficients (β) were exponentiated to calculate the multiplicative changes in SCRs. Multivariable‐adjusted models were adjusted for potential confounders, including the number of hospitals per 1000 km^2^, average monthly wage for ordinary workers, and university enrollment rate.

In the sensitivity analysis, linear regression analyses with log‐transformed SCRs as the dependent variable were used to assess robustness. Both unadjusted and multivariable‐adjusted regression models were used, including the same confounding factors as primary analysis. Additionally, the primary analysis was repeated after excluding the three prefectures lacking total outpatient claim number data.

A two‐sided *p* < 0.05 was considered statistically significant. All statistical analyses were performed using R software (version 4.4.3; R Foundation for Statistical Computing, Vienna, Austria) and Stata (version 16.1; StataCorp LLC, College Station, TX, USA).

## 3 Results

**FIGURE 1:**
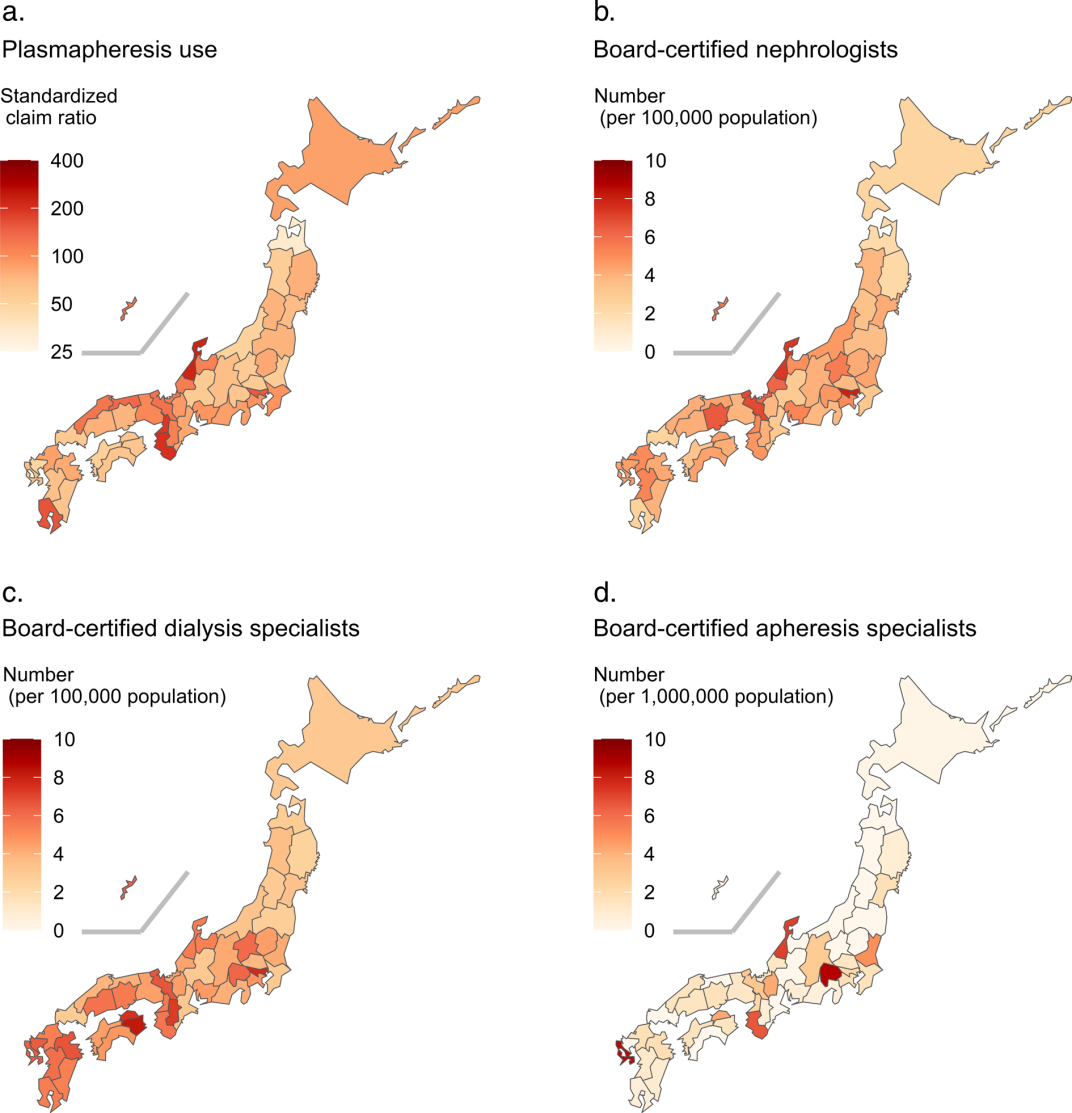
Regional variation in (a) plasmapheresis use and distribution of board‐certified (b) nephrologists, (c) dialysis specialists, and (d) apheresis specialists across Japan.

In fiscal year 2022, the number of claims for plasmapheresis was 23 888 for inpatients and 6706 for outpatients. The SCR for plasmapheresis ranged from 34.0 in Aomori to 222.4 in Ishikawa, representing a 6.5‐fold difference (Figure 1a). Median numbers of board‐certified nephrologists and dialysis specialists were 4.21 and 4.66 per 100 000 population, respectively, and that of apheresis specialists was 1.30 per 1 000 000 population (Figure 1b–d).

Prefectures with a higher density of board‐certified nephrologists had a greater number of hospitals (Table 1). Prefectures with a higher density of board‐certified dialysis specialists had a lower proportion of men and a higher hospital density (Table 2). Finally, prefectures with more board‐certified apheresis specialists had more hospitals, higher average monthly wages, and higher university enrollment rates (Table 3).

**TABLE 1:** Characteristics stratified by the number of board‐certified nephrologists.

	Number of board‐certified nephrologists (per 100 000 population)		*p*
	Low (2.16–4.21)	High (4.25–7.99)	
Number of prefectures	24	23	
Male proportion (%)	48.4 (47.4–49.1)	48.3 (47.4–49.1)	0.774
Proportion of population aged			
0–14 years (%)	11.4 (10.9–11.9)	11.8 (11.1–12.3)	0.282
15–64 years (%)	56.3 (54.3–58.1)	57.1 (54.9–59.3)	0.259
≥ 65 years (%)	32.6 (29.9–34.7)	31.2 (28.3–33.1)	0.148
Number of hospitals per 1000 km^2^	17 (9–24)	27 (18–46)	0.005
Average monthly wage (USD)	2158 (1986–2256)	2157 (2056–2322)	0.401
University enrollment rate (%)	53.8 (49.0–59.3)	56.4 (50.4–60.3)	0.268

*Note:* Data are presented as median and interquartile range.

Abbreviation: USD, United States dollars.

**TABLE 2:** Characteristics stratified by the number of board‐certified dialysis specialists.

	Number of board‐certified dialysis specialists (per 100 000 population)		*p*
	Low (2.54–4.66)	High (4.73–8.24)	
Number of prefectures	24	23	
Male proportion (%)	48.7 (47.7–49.4)	47.7 (47.3–48.7)	0.031
Proportion of population aged			
0–14 years (%)	11.4 (10.9–11.9)	11.8 (11.1–12.8)	0.179
15–64 years (%)	56.8 (54.8–58.5)	56.3 (54.3–58.9)	0.865
≥ 65 years (%)	31.9 (29.8–34.2)	31.5 (29.6–33.5)	0.609
Number of hospitals per 1000 km^2^	15 (9–23)	26 (20–39)	< 0.001
Average monthly wage (USD)	2159 (2020–2284)	2154 (2037–2256)	0.848
University enrollment rate (%)	53.8 (49.3–59.3)	56.5 (48.9–63.4)	0.259

*Note:* Data are presented as median and interquartile range.

Abbreviation: USD, United States dollars.

**TABLE 3:** Characteristics stratified by the number of board‐certified apheresis specialists.

	Number of board‐certified apheresis specialists (per 1 000 000 population)		*p*
	Low (0.00–1.30)	High (1.33–8.73)	
Number of prefectures	24	23	
Male proportion (%)	48.0 (47.3–49.1)	48.5 (47.7–49.1)	0.282
Proportion of population aged			
0–14 years (%)	11.4 (10.9–12.4)	11.5 (11.3–12.1)	0.516
15–64 years (%)	55.8 (54.5–57.8)	57.7 (54.8–60.0)	0.058
≥ 65 years (%)	32.8 (30.8–34.0)	30.4 (28.0–33.9)	0.051
Number of hospitals per 1000 km^2^	17 (9–25)	26 (18–56)	0.014
Average monthly wage (USD)	2047 (1959–2232)	2189 (2151–2322)	0.008
University enrollment rate (%)	50.6 (47.3–56.1)	57.8 (54.9–63.4)	< 0.001

*Note:* Data are presented as median and interquartile range.

Abbreviation: USD, United States dollars.

In unadjusted negative binomial regression models, the numbers of board‐certified nephrologists (*β* = 0.14; 95% confidence interval [CI]: 0.06 to 0.22; *p* = 0.001), dialysis specialists (*β* = 0.09; 95% CI: 0.01 to 0.17; *p* = 0.035), and apheresis specialists (*β* = 0.06; 95% CI: 0.01 to 0.11; *p* = 0.028) were significantly associated with SCRs for plasmapheresis. After adjustment for clinically relevant covariates, only the number of board‐certified nephrologists was significantly associated with the SCR for plasmapheresis (*β* = 0.11; 95% CI: 0.02 to 0.20; *p* = 0.022), whereas the numbers of board‐certified dialysis (*β* = 0.05; 95% CI: −0.04 to 0.13; *p* = 0.298) and apheresis specialists (*β* = 0.05; 95% CI: 0.00 to 0.10; *p* = 0.053) were not (Table 4). These findings indicated that each additional nephrologist per 100 000 population was associated with a 1.11‐fold increase in the SCR for plasmapheresis (*e* ^0.11^ = 1.11).

**TABLE 4:** Associations between the number of board‐certified specialists and plasmapheresis use.

		Number of board‐certified		
		Nephrologists (per 1/100 000 population)	Dialysis specialists (per 1/100 000 population)	Apheresis specialists (per 1/1 000 000 population)
Negative binomial regression model				
Number		47	47	47
Unadjusted	*β* (95% CI)	0.14 (0.06 to 0.22)	0.09 (0.01 to 0.17)	0.06 (0.01 to 0.11)
	*e* ^*β*^ (95% CI)	1.15 (1.06 to 1.24)	1.09 (1.01 to 1.18)	1.06 (1.01 to 1.12)
	*p*	0.001	0.035	0.028
Adjusted^a^	*β* (95% CI)	0.11 (0.02 to 0.20)	0.05 (−0.04 to 0.13)	0.05 (0.00 to 0.10)
	*e* ^*β*^ (95% CI)	1.11 (1.02 to 1.22)	1.05 (0.96 to 1.14)	1.05 (1.00 to 1.10)
	*p*	0.022	0.298	0.053
Linear regression model				
Number		47	47	47
Unadjusted	*β* (95% CI)	0.14 (0.06 to 0.23)	0.07 (−0.01 to 0.15)	0.04 (−0.01 to 0.09)
	*e* ^*β*^ (95% CI)	1.15 (1.06 to 1.26)	1.07 (0.99 to 1.16)	1.04 (0.99 to 1.10)
	*p*	0.002	0.095	0.119
Adjusted^a^	*β* (95% CI)	0.10 (0.00 to 0.21)	0.03 (−0.05 to 0.12)	0.03 (−0.02 to 0.08)
	*e* ^*β*^ (95% CI)	1.11 (1.01 to 1.23)	1.03 (0.95 to 1.13)	1.03 (0.98 to 1.09)
	*p*	0.043	0.429	0.228

Abbreviation: CI, confidence interval.

^a^ Adjusted for the number of hospitals per 1000 km^2^, average monthly wage for ordinary workers, and university enrollment rate.

Linear regression analyses showed similar associations. The number of board‐certified nephrologists remained significantly associated with plasmapheresis use (adjusted *β* = 0.10; 95% CI: 0.00 to 0.21; *p* = 0.043), whereas the numbers of board‐certified dialysis (adjusted *β* = 0.03; 95% CI: −0.05 to 0.12; *p* = 0.429) and apheresis specialists (adjusted *β* = 0.03; 95% CI: −0.02 to 0.08; *p* = 0.228) were not associated with plasmapheresis use (Table 4).

Similar associations were observed in the multivariable‐adjusted models excluding the three prefectures without total outpatient claim number data: the number of board‐certified nephrologists remained significantly associated with plasmapheresis use (adjusted *β* = 0.12; 95% CI: 0.03 to 0.21; *p* = 0.011). The numbers of board‐certified dialysis (adjusted ß = 0.04; 95% CI: −0.07 to 0.15; *p* = 0.459) and apheresis specialists (adjusted *β* = 0.04; 95% CI: −0.01 to 0.09; *p* = 0.145) were not significantly associated in these adjusted models (Table S1).

## 4 Discussion

This cross‐sectional study highlights regional variations in plasmapheresis use across Japan. The number of board‐certified nephrologists, but not dialysis and apheresis specialists, was positively associated with plasmapheresis use. These findings suggest that the presence and equitable regional distribution of board‐certified nephrologists may enhance access to plasmapheresis.

Although no prior study has simultaneously examined regional differences in plasmapheresis use and their association with specialist distribution, a United States cross‐sectional study reported significantly higher plasmapheresis use in the southern region than elsewhere [19, 20]. In Japan, studies on regional variations in clinical practice and specialist distribution have shown similar associations. In one cross‐sectional study, respiratory specialist density was associated with the SCR for benralizumab prescription for severe asthma [23]. Moreover, the number of board‐certified radiation oncologists was positively correlated with intensity‐modulated radiotherapy use for cancer [24]. Consistently, we identified regional variations in plasmapheresis use and a positive association with the number of board‐certified nephrologists.

A plausible explanation for this association is the availability of physicians trained to perform the procedure. Nephrologists typically conduct and manage plasmapheresis; therefore, they require comprehensive knowledge of its indications, prescription practices, appropriate replacement fluid selection, judicious anticoagulant use, and procedural complication monitoring and management [41, 42]. Notably, in a survey of physicians in Australia and New Zealand, 75% of nephrologists had easy access to plasmapheresis compared with 49% of other physicians [22]. Meanwhile, board‐certified dialysis and apheresis specialists include non‐nephrologists, who may have less access to performing plasmapheresis than nephrologists. Given that nephrologists generally have access to and expertise in performing plasmapheresis, regions with more board‐certified nephrologists may show greater plasmapheresis utilization. Additionally, the small number of apheresis specialists may have hindered meaningful statistical analysis, failing to reveal an association. In 2022, 5931 board‐certified nephrologists existed, whereas only 208 board‐certified apheresis specialists were present nationwide.

This study had some limitations. First, the use of prefecture‐level aggregated data allowed assessment of population‐level associations, which may not reflect relationships at the individual level. Consequently, the results should not be generalized to smaller geographic areas or individual patients within each prefecture. Second, the cross‐sectional design limited the ability to infer causality between the number of specialists and plasmapheresis use. Longitudinal or cohort studies are warranted to further assess potential causal associations. Third, the SCR for plasmapheresis may be biased, as data for age–sex groups with < 10 claims were withheld in the NDB Open Data and thus excluded from SCR calculation. Fourth, the NDB Open Data lacked information on indication‐specific diseases for plasmapheresis. Consequently, regional variations in plasmapheresis use according to specific indications or underlying diseases could not be evaluated.

## 5 Conclusions

This study revealed regional variations in plasmapheresis use in Japan, associated with the distribution of board‐certified nephrologists. Addressing the regional disparities in nephrologist availability could help reduce inequities in access to plasmapheresis, facilitate timely treatment for patients requiring urgent intervention, and promote more consistent, high‐quality patient care nationwide. Further research using well‐designed cohort studies is needed to clarify the factors underlying regional differences in plasmapheresis use.

## Author Contributions

R.Y., K.M., and M.E. contributed to the study concept, design, and organization. R.Y. performed data acquisition, management, and statistical analysis. K.M., K.Y., M.E., Y.I., and T.I. contributed to data interpretation. T.I. provided study supervision and mentorship. All coauthors contributed important intellectual input to manuscript drafting and revision. Moreover, all coauthors accept responsibility for the work and will ensure that any concerns regarding accuracy or integrity are investigated and resolved.

## Funding

The authors have nothing to report.

## Ethics Statement

This study was approved by the Ethics Committee of Izumo Medical Life Cooperative Izumo‐Shimin Hospital (approval number: 25‐01) and conducted in accordance with the principles of the Declaration of Helsinki.

## Consent

This study used an opt‐out approach to obtain informed consent according to the Japanese Ethical Guidelines for Medical and Health Research Involving Human Subjects.

## Conflicts of Interest

The authors declare no conflicts of interest.

## Supporting information


**Table S1:** Association between the number of board‐certified specialists and plasmapheresis use across 44 prefectures in Japan.

## Data Availability

NDB Open Data are publicly available via the Ministry of Health, Labour and Welfare. Data on the number of board‐certified nephrologists, dialysis specialists, and apheresis specialists in fiscal year 2022 were obtained from the Japanese Society of Nephrology, Japanese Society for Dialysis Therapy, and Japanese Society for Apheresis with permission and cannot be shared by the authors. Access to these data requires approval from the respective academic societies.
