# Supplementary material for: Association Between Specialist Distribution and Regional Variation in Plasmapheresis Use in Japan: A Population‐Level Cross‐Sectional Study
Source: J Clin Apher. 2026 Mar 15;41(2):e70112. doi: 10.1002/jca.70112 (PMC12989638; doi:10.1002/jca.70112)
Supplement: Supplementary file 1 — Table S1: Association between the number of board‐certified specialists and plasmapheresis use across 44 prefectures in Japan. [file JCA-41-e70112-s001.pdf]

Supplementary Table 1. Associations between the number of board-certified specialists and plasmapheresis use in 44 prefectures

		Number of board-certified		
		Nephrologists (per 1/100,000 population)	Dialysis specialists (per 1/100,000 population)	Apheresis specialists (per 1/1,000,000 population)
Negative binomial regression model				
Number		44	44	44
Unadjusted	ß (95% CI)	0.14 (0.06 to 0.22)	0.07 (−0.01 to 0.16)	0.05 (0.00 to 0.10)
	$e^{ß}$ (95% CI)	1.15 (1.06 to 1.24)	1.08 (0.99 to 1.17)	1.05 (1.00 to 1.11)
	p	0.001	0.086	0.062
Adjusted*	ß (95% CI)	0.12 (0.03 to 0.21)	0.04 (−0.07 to 0.15)	0.04 (−0.01 to 0.09)
	$e^{ß}$ (95% CI)	1.12 (1.03 to 1.23)	1.04 (0.94 to 1.16)	1.04 (0.99 to 1.10)
	p	0.011	0.459	0.145
Linear regression model				
Number		44	44	44
Unadjusted	ß (95% CI)	0.14 (0.05 to 0.23)	0.06 (−0.03 to 0.14)	0.03 (−0.02 to 0.09)
	$e^{ß}$ (95% CI)	1.15 (1.05 to 1.26)	1.06 (0.97 to 1.15)	1.03 (0.98 to 1.09)
	p	0.002	0.190	0.218
Adjusted*	ß (95% CI)	0.11 (0.01 to 0.22)	0.03 (−0.09 to 0.14)	0.02 (−0.03 to 0.08)
	$e^{ß}$ (95% CI)	1.12 (1.01 to 1.24)	1.03 (0.91 to 1.16)	1.02 (0.97 to 1.08)
	p	0.033	0.643	0.429

Abbreviation: CI, confidence interval

*Adjusted for the number of hospitals per 1,000,000 population, average monthly wage for ordinal workers, and university enrollment rate
